# Rotating Hinge Prosthesis for Primary and Revision Knee Arthroplasty: Comparison and Indications

**DOI:** 10.1155/2022/9930675

**Published:** 2022-01-30

**Authors:** Hans-Joachim Neuhaus, Kristin Maier

**Affiliations:** ^1^Department of Traumatology and Orthopaedics, St. Vincenz Hospital, 58706 Menden, Germany; ^2^Aesculap AG, Medical Scientific Affairs, 78532 Tuttlingen, Germany

## Abstract

**Background:**

Rotating hinge knee prostheses are typically used in revision and severe primary total knee arthroplasty (TKA). For these challenging patient groups, currently only few studies with mid- or even long-term follow-up and adequate patient numbers are available. In addition, a more specific definition is needed of the indications for a rotating hinge prothesis in primary patients beyond the use in bone defects.

**Methods:**

In this prospective study, 170 primary and 62 revision TKA patients were included who received a rotating hinge knee prosthesis at the study centre between the years 2009 and 2014. Of these, 98 primary and 22 revision TKA patients were available for 5-year functional and clinical follow-up examinations. Prosthesis survival in both patient groups could be compared up to a 9-year follow-up.

**Results:**

Postoperatively, functional results including range of motion (ROM) and clinical scores like the Oxford Knee Score (OKS) and subscales of the Knee Society Score (KSS) improved better in patients treated for primary knee arthroplasty than for revision patients. Besides the patient group (primary vs. revision TKA), no overall influencing factors (age, body mass index, gender, etc.) regarding functional results could be identified in a multiple linear regression analysis. The revision rate of primary patients was significantly lower than in the revision patients, with an 8-year Kaplan-Meier prosthesis survival of 88% in the Primary and 60% in the Revision group.

**Conclusion:**

The prosthesis provides promising results in severe primary and revision knee arthroplasty. In addition to commonly agreed recommendations regarding the use of rotating hinge knee prostheses for primary surgery, six specific indications are proposed and discussed here as a base for scientific debate.

## 1. Introduction

Total knee arthroplasty (TKA) is a frequently performed surgery with an increasing number of interventions [1, 2]. The aim of this intervention is the restoration of knee function and reduction of pain. Performance and survival of the TKA prostheses accomplished positive development in the past, but due to the increasing number of patients with such implants, the number of TKA revisions is also increasing [1, 2]. Bone loss, ligament instability, and poor condition of the periprosthetic soft tissue make revision difficult, requiring more constrained prosthesis types [3–5]. Rotating hinge prostheses were developed to address these issues. These comprise a hinge mechanism, coupling the femoral and the tibial component and facilitating flexion-extension movement. Current “Third generation” rotating hinge prostheses also allow axial rotation and distraction. Femoral and tibial components are typically fixed by an intramedullary stem, and elements to compensate bony defects may be added [6–9].

In the beginning, hinged knee prostheses were only designated for severe revision cases and salvage therapy. Today, the deployment of a rotating hinge prosthesis also in primary TKA is a matter of debate amongst the orthopaedic society [10, 11]. Conservative indications for primary TKA with rotating hinge prostheses comprise massive bone defects, including femoral or tibial bone tumour resection, and severely damaged ligaments. However, some authors propose a wider field of indications [10, 12]. Results of rotating hinge prostheses in primary and revision TKA are rather difficult to distinguish and interpret due to the low number of published studies and limiting factors including study design, different prosthesis systems, and short follow-up period.

In this publication, functional and survival results of the EnduRo rotating hinge prosthesis are presented from a prospective patient cohort. The hypothesis was that EnduRo obtains reliable results in primary and revision TKA patients. Short-term results of a part of this patient cohort and a second study centre were already published by Giurea et al. [13], when a total number of 62 patients had reached the two-year follow-up. While the clinical results were very promising, significant differences in functional outcome and risk of reoperation in the Primary and Revision groups were already observed. The present study continues the analysis with a much larger monocentric patient cohort and with a midterm follow-up of 5 years.

## 2. Materials and Methods

### 2.1. Study Population

**Figure 1:**
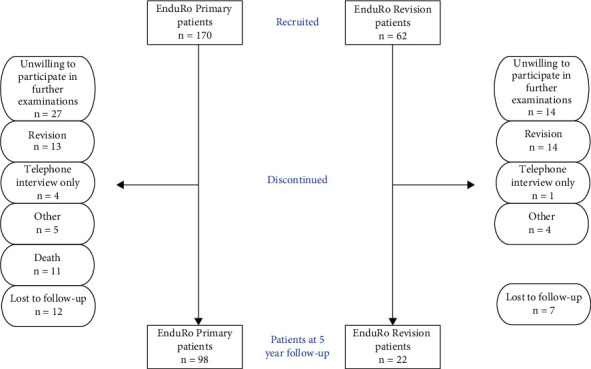
Patient inclusion flowchart. The flowchart describes the recruitment, reasons for discontinuation, and number of patients at the 5-year functional follow-up examination. The number of revisions resulting in discontinuation of the study includes revisions with and without component exchange.

This study was a prospective single centre study, performed according to the Declaration of Helsinki. Eligibility criteria were limited to patients in the clinic of the principal investigator treated with the EnduRo rotating hinge prosthesis for primary or revision TKA for all indications except bone tumours. Between March 2009 and February 2014, 290 patients were treated with the prosthesis in the clinic of the principal investigator; thereof 232 patients could be included into the study. Patients were contacted annually for follow-up examinations (either physically or by phone, as described in Follow-Up and Data Collection). At time of the 5-year follow-up, the number of patients discontinuing the study due to withdrawal, revision, loss to follow-up, death, and other reasons was 72 patients in the Primary group and 40 patients in the Revision group (see Figure 1). At study finalization, 98 patients were still included in the Primary group, and 22 patients were included in the Revision group.

### 2.2. Surgical Procedure

The modular rotating hinge prosthesis system EnduRo (Aesculap AG, Tuttlingen, Germany) was used in all patients. The hinge mechanism is not primary weight bearing as force is transmitted from the femoral component to the tibial component via the polyethylene insert with high contact area. An offset option and wedges for the tibial and femoral components are available to cover specific anatomical characteristics. As a unique feature, EnduRo contains flanges and bushings that are made of carbon-fibre reinforced poly-ether-ether-ketone (CFR-PEEK) [13].

All surgeries were performed by four orthopaedic expert surgeons; the majority (91 of 120 patients) was performed by the principal investigator (Hans-Joachim Neuhaus). Surgeries were performed according to the procedure described by Giurea et al., and the method description partly reproduces their wording [14]. Briefly, all surgeries were performed through a medial parapatellar arthrotomy. Cefazolin (3 × 2 g) or Clindamycin (3 × 600 mg) was used for perioperative antibiotic prophylaxis, and low-molecular weight heparin (40 mg–60 mg/day) was used for thrombotic prophylaxis starting 12 hours before surgery continuing for 6 weeks postoperatively. For implantation, the hybrid stem technique with cemented femoral and tibial epiphyseal fixation and uncemented stems was performed. Postoperatively, crutches or a walker could be used by the patients if needed, and partial to full weight bearing was allowed as tolerated. Functional and radiographic examination was performed at discharge and after 3 months.

### 2.3. Follow-Up and Data Collection

In addition to the documentation of baseline demographic data, medical history, and perioperative assessment, a yearly follow-up of the patients was carried out, which is at time of this report still ongoing until a planned physical follow-up examination after 10 years.

Physical examinations were performed at the time points 1, 2, and 5 years in the outpatient department of the principal investigator's clinic. All other yearly follow-up time points were conducted by telephone interview only. The 5 year follow-up was completed in 2019. Primary outcomes obtained during follow-up were functional and clinical results. These were assessed using the knee (kKSS) and functional (fKSS) KSS subscales [15], the OKS [16], and ROM. ROM was measured using a goniometer, and a possible overflexion was not incorporated into the measurement.

The revision rate was defined as secondary outcome variable. Complications resulting in revisions were categorized into 3 types according to Giurea et al. and Böhler et al. [14, 17]. Type 1 complications were defined as infection; type 2 as periprosthetic complications such as periprosthetic fracture, extensor mechanism failure, patella problems, and wound healing disturbances; and type 3 complications as implant complications such as aseptic loosening, wear, prosthesis failure (fracture of axis, bushings, and stem), and instability. Type 1 and type 2 complications are associated with minor impact from the prosthesis itself, whereas type 3 complications are directly induced or strongly associated with the prosthesis.

Survival data (implantation status, reason for and time of revision, time of censoring) were collected to the best of our knowledge, also in case of deceased patients or patients who were not able or not willing to participate in the yearly phone interviews. All survival-related data until database lock in 2019 were included into revision analysis and Kaplan-Meier calculation. Ethical approval was received from the regional institutional review board, and all patients provided written informed consent.

### 2.4. Statistical Analysis

Statistical analysis was performed using the SAS software version 9.4 (SAS Inc., Chicago, IL). Patients with completed 5-year follow-up were included in the analysis of functional and clinical outcome. *t*-tests were used for comparison of continuous outcomes between groups; differences were considered significant at *p* values below 0.05. As all analyses were explorative, this level does not apply in a confirmative sense, and no multiplicity adjustments were made.

Kaplan-Meier method was used for analysis of the prosthesis survival rate; point-wise 95% confidence intervals were calculated according to Agresti and Coull [18]. Patients discontinuing the study with prothesis in situ were included as censored events. In case of lost to follow-up patients, the date of the last known implant status was used as time point of the censoring event. For the comparison of patient groups, a log-rank test was conducted with significance level of *p* = 0.05. All survival-related data which were available until the time of completion of the 5-year follow-up were included for calculation of survival rates.

Multiple linear regression repeated measure models with prespecified set of impacting variables were used to analyse the risks for continuous outcomes. The variable set consisted of age, gender, BMI, baseline score, visit ID, and patient group (primary vs. revision TKA).

## 3. Results

### 3.1. Patient Characteristics and Indications

Of the 170 primary and 62 revision TKA patients which were prospectively included in the study, 98 primary and 22 revision TKA patients were still available for the 5-year follow-up examinations. For the analysis of survival rates, additionally data from the yearly telephone follow-up time points of up to 9 years were used.

Patient characteristics of both groups are provided in Table 1. The mean age, operation time, hospital stay, and proportion of female patients were higher in the Revision group, whereas BMI was comparable.

Table 2 shows the main indication for a rotating hinge prosthesis in the Primary group. Gonarthrosis was present in all patients. The main indication for a rotating hinge prosthesis was varus/valgus malalignment in 43.9% of the cases, followed by ligamentous instability (21.4%), instability and malalignment (11.2%), and others such as extension deficit, obesity, posttraumatic gonarthrosis, rheumatism, or bone loss. Two patients were intraoperatively switched to rotating hinge prosthesis after the planned unconstrained bicondylar prothesis could not be balanced.

The main indication in the Revision group (Table 3) was instability (54.5%) and aseptic loosening (13.6%). Further indications included periprosthetic fracture, arthrofibrosis, and other reasons. Primary prostheses in the Revision group were unconstrained bicondylar TKA (72.7%) and unicompartmental knee prostheses (27.2%).

### 3.2. Functional and Clinical Results

**Figure 2:**
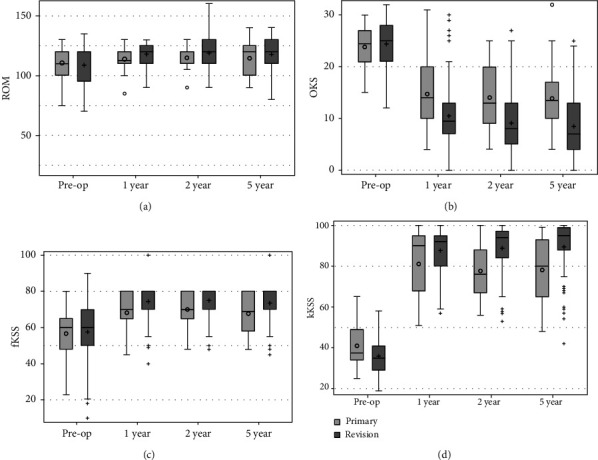
Functional results of the Primary (light grey) and Revision groups (dark grey) at different follow-up points. (a) ROM is provided in °. (b) OKS, (c) fKSS, and (d) kKSS are given in points.

The performance of the prosthesis was assessed using ROM, functional, and clinical scores. Results of the Primary and Revision groups in terms of ROM, OKS, fKSS, and kKSS are provided in Figure 2. A significant improvement between the preoperative and postoperative values was obtained for ROM and all scores. Mean ROM improved from preoperative 108.7° and 110.7° in the Primary and Revision group to 117.9° and 114.6° 5 years postoperatively. In the Primary and Revision groups, the mean OKS improved from 24.4 and 23.8 points preoperatively to 8.5 and 13.9 points 5 years postoperatively. Preoperative mean fKSS of the Primary and Revision group were 57.3 and 56.7 points, improving to 73.5 and 67.8 points 5 years postoperatively. For kKSS, an improvement from preoperative 35.0 and 37.5 points to 89.5 and 78.1 points 5 years postoperatively was obtained in the Primary and Revision group.

No statistically significant difference was present between Primary and Revision groups for preoperative ROM, OKS, and kKSS, whereas preoperative fKSS was significantly lower in the Revision group compared to the Primary group. Postoperatively, ROM was significantly higher in the Primary group only 1 year postoperatively, whereas all other scores were significantly higher in the Primary group in comparison to the Revision group (*p* ≤ 0.05) at all time points. Conclusively, compared to the preoperative state, the implantation of EnduRo achieved an improvement of ROM, OKS, kKSS, and fKSS values, with better results in the Primary than in the Revision group.

### 3.3. Influencing Factors

To investigate whether distinct factors may influence postoperative functional and clinical results, an analysis of impacting variables (covariates and factors) was performed (see Table 4). In addition to the patient group (primary vs. revision TKA), which was identified as an influencing factor for all scores, only the baseline score had a significant impact in terms of ROM. For OKS, baseline score, visit ID, and higher BMI had a significant impact on the results. Specifically, a higher BMI was predictive of inferior postoperative OKS values. For fKSS, higher age had a negative impact on postoperative values. Regarding kKSS, no influencing factor other than patient group was identified.

### 3.4. Revisions

Revision was defined as exchange of one or more prosthesis components, excluding patella resurfacing due to secondary retropatellar arthrosis and prophylactic exchange of polyethylene components. At database lock in 2019, which corresponds to a mean follow-up of 57.1 and 43.7 months of the Primary and Revision group, 15 primary patients (9.7% of the initial patient population) and 13 revision patients (20%) had to be revised (see Table 5).

Complications resulting in revision were categorized into type 1, infection; type 2, periprosthetic complications (e.g., periprosthetic fracture, extensor mechanism failure, patella problems, and wound healing disturbances); and type 3, implant complications (e.g., aseptic loosening, wear, prosthesis failure, and instability).

The most frequent reason for revision in the Primary group was infection (7 patients) and patella-related problems (4 patients). Type 3 complications in the Primary group included aseptic loosening and instability (1 patient each).

In the Revision group, six patients were revised due to infection, six patients were revised due to aseptic loosening, and one patient was revised due to poor function.

### 3.5. Survival

**Figure 3:**
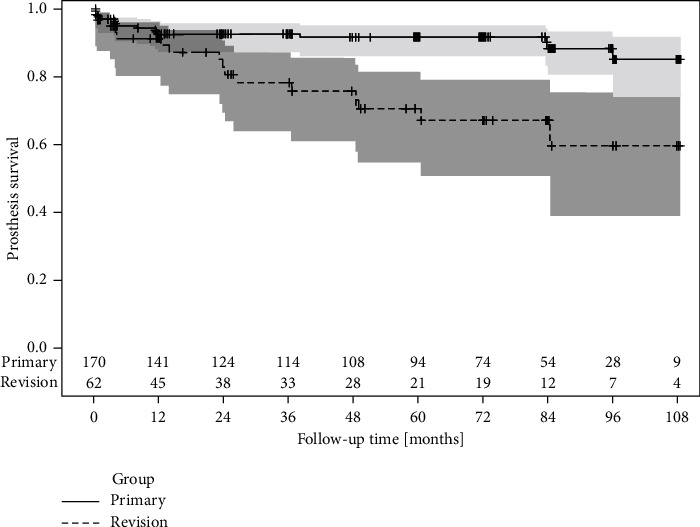
Kaplan-Meier revision free survival of the prosthesis in the Primary and Revision group, with 95% confidence intervals. The number of patients at risk is shown on the bottom line.

Kaplan-Meier analysis was performed to assess survival of the prosthesis in the Primary and Revision group. Survival probabilities after 1 year in the Primary and Revision group were 93% and 91%, respectively (see Figure 3). Values for 2, 5, and 8 years were 93%, 91%, and 88% in the Primary group and 83%, 70%, and 60% in the Revision group. Medium-term prosthesis survival was significantly higher in the Primary group compared to the Revision group (*p* = 0.0004).

## 4. Discussion

### 4.1. Functional and Clinical Results

The most important finding of the current study is that the EnduRo rotating hinge system achieves good results in primary and revision TKA. Comparison between the Primary and Revision group showed better postoperative functional results and survival rates in the Primary group.

Böhler et al. [17] presented medium-term results from the second study centre of the publication by Giurea et al. [14]. In contrast to the results of the current study, significantly better results were obtained in the Primary group only for kKSS, whereas no differences were observed for fKSS, WOMAC score, OKS, and ROM. Prosthesis survival was significantly lower in the Revision group than in the Primary group, which is consistent with the results from the current study. Logistic regression indicated revision surgery and age as influencing risk factors for complications [17].

Ochs et al. presented the results of primary and revision TKA with EnduRo, which was implanted using a navigation system [19]. In this study, significantly better kKSS values and ROM were obtained in the Primary group than in the Revision group, whereas no differences were observed for fKSS [19].

Further studies directly comparing the functional results of rotating hinge prostheses in primary and revision TKA are rare and based on small patient cohorts. Efe et al. found no significant differences of kKSS, fKSS, and ROM in primary and revision TKA treated with Endo-Modell (Waldemar Link, Hamburg, Germany). However, there were clearly more complications and a lower survival rate in the Revision group [20]. In another study comparing the results of primary and revision TKA with the same prosthesis, also no differences were observed regarding kKSS, fKSS, ROM, and survival rate [21].

### 4.2. Revisions, Complications, and Survival Rate

In the current study, the revision rate of the Revision group was about twice as much as that of the Primary group. In the Primary group, the most prevalent type of complications was infection, followed type 2: periprosthetic complications, especially patella problems. Type 1 and type 2 complications are not directly associated with the prosthesis itself but rather with the surgical technique and tissue quality [14, 17].

Revision was necessary in 9.7% of the Primary group patients and 20% of the Revision group patients after 9 years. Failure probability of hinged knee prostheses in the EPRD after 4 years is just below 7%, whereas for standard nonconstrained TKA it is about 3.5% [22]. The Australian Orthopaedic Association National Joint Replacement Registry (NJRR) reports 8.4% revisions after 5 and 12.7% after 10 years for hinged knee prostheses, whereas these rates for posterior stabilized TKA are 4% and 6% [23]. In the National Joint Registry of England, Wales, Northern Ireland and the Isle of Man (NJR), 5- and 10-year revision rates for hinged knee prostheses are 6.1% and 9.7%, whereas those rates for unconstrained knee prostheses are between 2 and 4.2% [24].

In the study by Efe et al. with a mean follow-up of 56 months of 21 primary TKA and 28 revision TKA cases with rotating hinge prothesis Endo-Modell (Waldemar Link), (re-)revision rates of 5% in primary and 24% in revision TKA were reported [20]. Likewise, after a follow-up of 5 years, 1 of 24 patients (4.2%) treated with EnduRo for primary TKA was revised in the study by Böhler et al. whereas 10 of 26 patients (41.7%) treated with revision surgery were re-revised [17]. Hintze et al. reported 15 prosthesis revisions (12%) in 125 revision TKA treated with NexGen RHK (Zimmer Biomet, Warsaw, IN, USA) after a follow-up of 6.2 years [25]. In a retrospective study of Pfeufer et al. with the same prosthesis, 16.6% implant revisions (8 complete or partial component revisions) were reported in 48 primary and revision TKA procedures after a mean follow-up of 2.6 years [11]. Regarding revision rates, our results are in the range of rotating hinge prostheses used for primary and revision surgery reported in national joint registries and scientific publications.

In the current study, the major reasons for revision in the Primary group were infection and patella problems, whereas re-revision in the Revision group was mostly necessary due to infection and aseptic loosening. In the EPRD, aseptic loosening and infection are the most prevalent causes for revision of primary TKA with 24% and 14.5% [22]. Likewise, aseptic loosening (NJRR: revision: 24.7%, re-revision: 33.3%; NJR: revision: 38.7%, re-revision: 33.9%) and infection (NJRR: revision: 23.7%, re-revision: 25.1%; NJR: revision: 18.3%, re-revision: 23.1%) are the most common causes for revision and re-revision in the NJRR and NJR [23, 24]. Further publications identified infection, aseptic loosening, instability, and stiffness as main reasons for revision and re-revision of TKA [26–29]. Therefore, besides patella problems, the revision causes in the current cohort are in line with those from national joint registries and other studies. The rather high revision rate due to patella problems in the current cohort may be attributable to a lower rate of patella resurfacing in the beginning of the application of this prosthesis in our clinic. After the initial experience of a rather high number of revisions due to patella problems, the share of interventions including patella resurfacing increased, and revisions due to patella problems were reduced. A similar trend of increasing use of a patella component in primary TKA can also be observed in the EPRD and NJRR [6, 7], whereas the Swedish joint registry recorded decreasing rates of patellar resurfacing [13]. The issue with patella problems demonstrates the impact of a learning curve regarding the application of a new implant system, which we also recognized in our experience with this prothesis.

Another important point regarding the learning curve concerns the preoperative planning of the tibia and femur stems. During this critical step, it has to be assessed whether the scheduled stem fits the intramedullary canal. Especially in older patients, the bone may be deformed, resulting in a curved intramedullary canal. In these cases, a too long stem would make excessive contact with the cortical bone, inducing high levels of stress and bone remodelling, which can eventually cause aseptic loosening. If preoperative planning shows that a cementless stem cannot be implanted without too much contact with the cortical bone, a short cemented stem is chosen. This stem is implanted eccentric in a large diameter hole, which is filled with bone cement to prevent cortical bone overstressing.

In the study patients, no case of loosening due to shearing forces on the prosthesis was observed. This may be attributed to our surgical technique regarding the collateral ligaments: We do not trim the collateral ligaments but rather peel them from the femoral epicondyle before implanting the prosthesis. Afterwards, the collateral ligaments place back into position on the epicondyles and grow onto them (which we see in patients where early reoperations were required). Our hypothesis is that the ongrown collateral ligaments partially gain back function and therefore reduce shearing forces on the prosthesis. This technique and the potential partial recovery of function of the collateral ligaments may contribute to the improvement of functional results up to the two-year follow-up.

### 4.3. Limitations

This study has some limitations. First, there was a high number of patients who dropped out of the study, and some of the patients did not participate in all follow-up examinations. However, the number of included patients was sufficient for statistical analysis. If possible, the implantation status of patients who were not able or not willing to participate and of deceased patients was documented for the calculation of survival. However, the number of revisions may still be underestimated. Second, the period in which the index surgeries were performed is rather long. Therefore, the effects of a learning curve are included in this study. Third, the reasons for revision surgery are very specific and may have an influence on the results in the Revision group. However, from a statistical perspective, it was not reasonable to study this influence. Fourth, the study was performed with a specific rotating hinge prosthesis brand, and the transferability of the results to other rotating hinge knee systems in general has to be proven.

### 4.4. Indications for Primary TKA with a Rotating Hinge Implant in the Clinic of the Principal Investigator

Although the EnduRo prosthesis is intended for revision surgery or primary TKA with bone defects, in our clinic, it is frequently used also for primary TKA with specific indications. These include excessive varus (≥20°) and valgus (≥15°) deformities and ligamentous instability, especially instability of the medial collateral ligament, which is in line with current publications [9, 30].

Further specific indications are proposed from the point of view of the first author and presented below:

- *Insufficient Posterior Cruciate Ligament*. During preparation of the tibia for a cruciate-retaining prosthesis, there is a distinct risk of injuring the root of the ligament, especially with increasing tibial slope [31]
- *Severe Extension or Flexion Deficit (≥20°)*. Extension deficits may be induced by fibrotic changes of the dorsal capsule [32]. In such cases, release of the dorsal capsule and implantation of a rotating hinge prosthesis are performed to obtain higher ROM and sufficient stability
- *Knees Designated for Unconstrained TKA, Which Cannot Be Balanced Intraoperatively by Navigation Technology*. Intraoperative switch to a rotating hinge prosthesis is performed to achieve a well-stabilized postoperative result
- *Rheumatic Patients*. Bone and soft tissue quality is often compromised, and ligaments are frequently insufficient, so that stability can only be achieved via constrained prostheses on the long term
- *Patients with Neurologic Disorders, Especially Spasticity*. The need for TKA often derives from an inability to stabilize the joint; therefore, more constrained prostheses shall be used from our point of view to obtain sufficient stability, especially on the long term
- *Obese Patients*. It was shown that in flexion extreme forces are transmitted to the distal femoral bone, which may result in loosening at the bone-implant interface [33, 34]. A rotating hinge system reflects a valuable alternative to unconstrained TKA for these patients in our opinion

We aim to highlight our specific views as a base for scientific debate. It has to be considered that rotating hinge TKA has higher complication and lower survival rates in comparison to unconstrained TKA. Furthermore, revision of a rotating hinge prosthesis is more challenging than revision of unconstrained TKA. Therefore, the benefits and potential disadvantages of such a treatment have to be weighed carefully for each patient, and preoperative planning has to be accomplished accurately. In all cases, the least invasive option of the rotating hinge prosthesis system is chosen, with short stems and cementless fixation with rather gliding-fit than press-fit anchorage.

## 5. Conclusions

To the best of our knowledge, the current study includes the largest patient cohort at a 5-year follow-up for the direct comparison of rotating hinge prostheses for primary and revision TKA regarding functional results. In addition, survival data are presented with a maximum follow-up of nine years. Therefore, this study has an impact on the scientific assessment of the controversy topic of rotating hinge knee prostheses for primary TKA.

In conclusion, the rotating hinge prosthesis system provides good medium-term results in severe primary and revision TKA. Functional results significantly improved compared to the preoperative state. In primary TKA patients, significantly better functional results and survival rates were obtained than in revision patients. Specific indications for primary TKA with rotating hinge prostheses are presented and act as a base for scientific discussion.

## Figures and Tables

**Table 1:** Patient characteristics.

	Primary	Revision
*N*	98	22
Age (mean) (SD)	68.3 years (±9.3)	71.4 years (±8.4)
Gender (female)	61.2%	72.7%
BMI (mean) (SD)	30.9 (±6.2)	29.9 (±3.9)
Operation time (mean) (SD)	127.6 min (±25.9)	153.5 min (±50.6)
Hospital stay (mean) (SD)	12.2 days (±4.1)	13.1 days (±3.3)

**Table 2:** Main indication in the Primary group.

Indication	*N*	%
Varus/valgus malalignment	43	43.9
Instability	21	21.4
Instability and varus/valgus malalignment	11	11.2
Extension deficit	7	7.1
Obesity	4	4.1
Posttraumatic gonarthrosis	4	4.1
Rheumatism	2	2.0
Unconstrained knee cannot be balanced	2	2.0
Bone loss	2	2.0
Other	2	2.0

**Table 3:** Reasons for revision in the Revision group.

Indication	*N*	%
Instability	12	54.5
Aseptic loosening	3	13.6
Periprosthetic fracture	1	4.5
Arthrofibrosis	1	4.5
Other	5	22.7

**Table 4:** Analysis of impacting values for ROM, OKS, fKSS, and kKSS. Due to the *p* value below 0.05, grey shaded fields are defined as influencing factors.

Effect	ROM	OKS	fKSS	kKSS
	*p* value			
Group	0.0155	0.0183	0.0014	0.0003
Baseline score	0.0008	0.0462	0.0758	0.2302
Visit ID	0.2161	0.0268	0.2209	0.3032
BMI	0.1572	0.0329	0.0540	0.7666
Gender	0.7961	0.4219	0.5314	0.3219
Age	0.4664	0.1385	0.0332	0.4514

**Table 5:** Revisions in the Primary and Revision group.

	Primary (*N*, %)	Revision (*N*, %)
Revisions	15, 9.7%	13, 20%
Type 1 complications	7, 46.7%	6, 46.2%
Type 2 complications	6, 40%	0
Type 3 complications	2, 13.3%	7, 53.8%

## Data Availability

The pre- and postoperative data used to support the findings of this study are available from the corresponding author upon request.
